# Supplementary figures and images for: Caution Is Needed When Using Niche Models to Infer Changes in Species Abundance: The Case of Two Sympatric Raptor Populations
Source: Animals (Basel). 2021 Jul 6;11(7):2020. doi: 10.3390/ani11072020 (PMC8300138; doi:10.3390/ani11072020)

**1997**

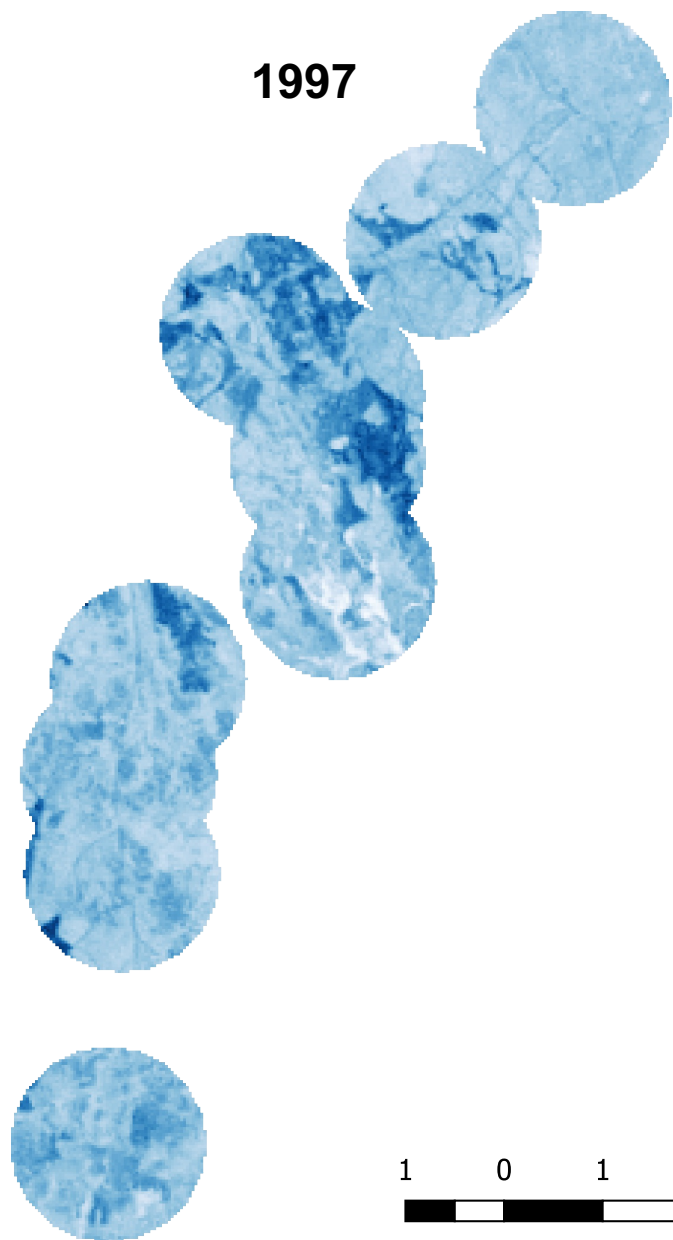

**2017**

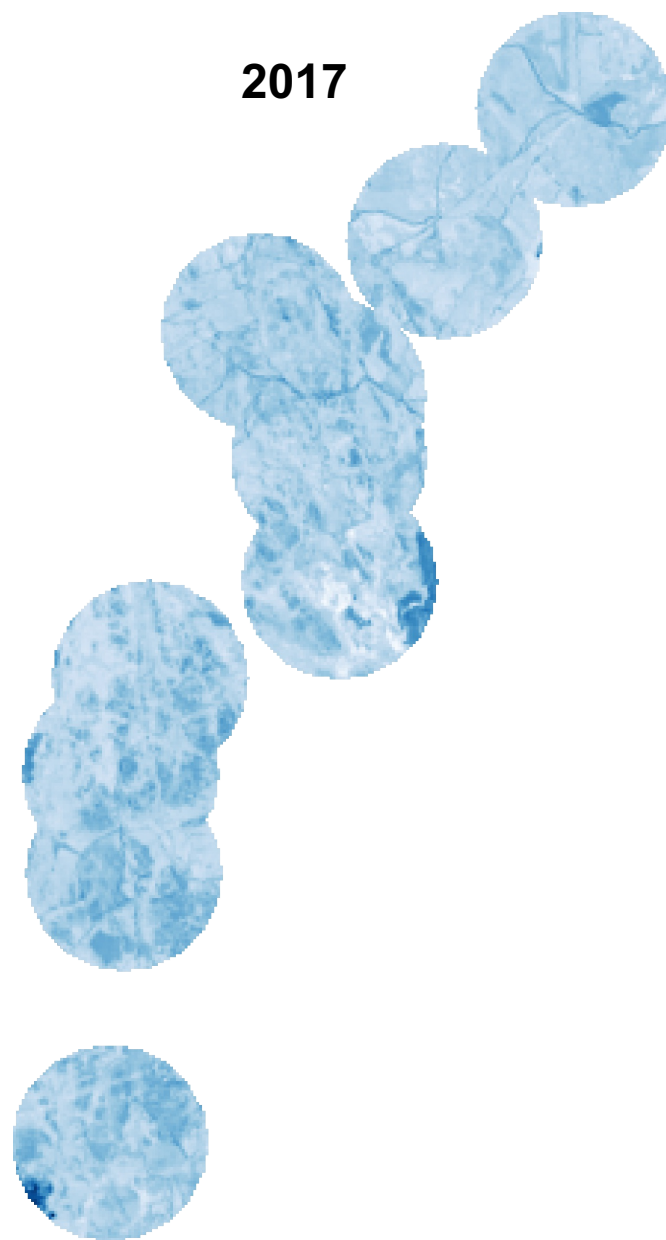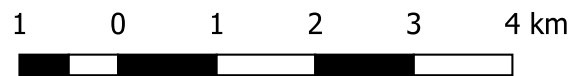

Supplement: Supplementary file 1 [file animals-11-02020-s001.zip › FiguraS1.pdf]
